# Moving Target Detection Using Dynamic Mode Decomposition

**DOI:** 10.3390/s18103461

**Published:** 2018-10-15

**Authors:** Jingwei Yin, Bing Liu, Guangping Zhu, Zhinan Xie

**Affiliations:** 1 Acoustic Science and Technology Laboratory, Harbin Engineering University, Harbin 150001, China; yinjingwei@hrbeu.edu.cn (J.Y.); liubing09513@hrbeu.edu.cn (B.L.); xiezhinan1984@gmail.com or xiezhinan@hrbeu.edu.cn (Z.X.); 2 Key Laboratory of Marine Information Acquisition and Security (Harbin Engineering University), Ministry of Industry and Information Technology, Harbin 150001, China; 3 College of Underwater Acoustic Engineering, Harbin Engineering University, Harbin 150001, China

**Keywords:** moving target detection, reverberation, dynamic mode decomposition

## Abstract

It is challenging to detect a moving target in the reverberant environment for a long time. In recent years, a kind of method based on low-rank and sparse theory was developed to study this problem. The multiframe data containing the target echo and reverberation are arranged in a matrix, and then, the detection is achieved by low-rank and sparse decomposition of the data matrix. In this paper, we introduce a new method for the matrix decomposition using dynamic mode decomposition (DMD). DMD is usually used to calculate eigenmodes of an approximate linear model. We divided the eigenmodes into two categories to realize low-rank and sparse decomposition such that we detected the target from the sparse component. Compared with the previous methods based on low-rank and sparse theory, our method improves the computation speed by approximately 4–90-times at the expense of a slight loss of detection gain. The efficient method has a big advantage for real-time processing. This method can spare time for other stages of processing to improve the detection performance. We have validated the method with three sets of underwater acoustic data.

## 1. Introduction

In addition to the noise of the marine environment and the self-noise of ships, an active sonar is usually disturbed by reverberation. Reverberation is the primary interference that limits the performance of active sonar detection in many cases. The interference of reverberation is particularly prominent in shallow water, which hinders efforts to detect weak targets, such as frogmen and small unmanned underwater vehicles. Reverberation is hard to remove, as it is strongly correlated with transmitted signals. In some cases, reverberation may even overwhelm the target echo in target detection for active sonar.

Many researchers have been attempting to suppress reverberation to detect moving targets for a long time. A method using autoregressive prewhiteners was first developed by Steven Kay et al. to suppress reverberation [1]. This method’s core idea is to build an autoregressive filter whose coefficients are estimated by processing a small amount of reverberation data. Andrea Trucco et al. improved the method by estimating the coefficients with a higher-order statistics algorithm [2]. Hichem Besbes and Sofia Ben Jebara used the normalized least mean squares algorithm to improve system identification [3] and used a variable step algorithm to reduce the computational complexity [4]. Li Wei et al. used the two-dimensional autoregressive algorithm to achieve good results in the case of a low signal-to-noise ratio [5]. Ashkan Tashk and Shapoor Khorshidi used data partitioning to select the order of the autoregressive prewhitening filter [6]. As a result, the deficiencies of the former prewhitening methods, such as high computational complexity or additional requirements for post-processing, were overcome. These methods are based on the assumption that the reverberation is stationary. However, the assumption is unsuitable in many cases. In addition, some of the methods also require some prior knowledge, such as the reverberation scattering function, but prior knowledge is difficult to obtain accurately.

Another kind of method is based on the linear frequency modulation (LFM) signal. The Wigner–Ville distribution (WVD) is a method of time-frequency conversion. This method can be used to separate the target from reverberation in the time-frequency domain, exploiting the characteristics of the LFM signal. S.Barbarossa and A. Zanalda showed that the combination of the WVD and the Hough transform is an important tool for mapping the signals onto a parameter space [7]. Peng-Lang Shui et al. processed two adjacent received signals using a cross-smoothed-pseudo-WVD and detected the target utilizing the features of the two results [8]. Yun-long Xia et al. added the processing of the time reversal mirror before WVD to suppress multiscattering [9]. These researchers obtained good results from the simulation. Although WVD represents the energy distribution of the signal well, the effect of the cross-terms still restricts the detection performance. A method was put forward by Yushuang Wu and Xiukun Li to remove the cross-terms in WVD [10], but it has not been applied for target detection. The energy of the LFM signal can be focused by fractional Fourier transform (FrFT), which is used for radar detection in heavy sea clutter [11,12]. Ge Yu et al. used FrFT in sonar detection and discussed the optimal transform angle of FrFT and the delay time estimation of the received signals in detail [13], whereas the disadvantage of FrFT is that the fractional order is difficult to choose.

Subspace-based techniques can be effectively used for target detection. Principal component inversion (PCI) is an analysis method for rapid adaptive signal detection [14,15]. Guillaume Ginolhac et al. considered reverberation to be the sum of echoes issued from the transmitted signal and then used PCI to estimate and remove the reverberation [16]. Dominant mode rejection (DMR) is another effective method for subspace analysis. Douglas A. Abraham and Norman L. Owsley used it to reduce the dimension space of the data matrix such that the target can be detected by dealing with low dimension space [16]. Subspace-based techniques attempt to separate the target from reverberation according to the orthogonality. However, the orthogonality is not always an adequate representation for data with a complex dynamic structure.

The development of low-rank and sparse theory [17,18,19,20] provides a new idea for moving target detection. Combining multiframe data, the moving target echo can be separated from the reverberation background, which takes advantage of the potential low-rank structure of reverberation. Based on this idea, Weichang Li et al. used a random projection algorithm for target tracking in the reverberant environment [21]. As an extension of their work, Feng-Xiang Ge et al. used convex optimization to decompose the data matrix instead of random projection and obtained better results [22]. However, the method increases the computational complexity, which is not conducive to the processing of data in real time.

In this paper, we introduce a new method to detect the moving target in a strong reverberant environment. We use dynamic mode decomposition (DMD) to decompose the data matrix, which is different from the two methods mentioned above. Our objective is to increase the speed of the target detection method dramatically without significant degradation of the detection gain. Our major contribution has the following three points. First, we restate the DMD from the perspective of the matrix decomposition, which can be regarded as an extension of [23]. Second, we divide the eigenmodes from DMD into two categories to realize the low-rank and sparse decomposition. Third, we apply DMD for target detection such that the computation speed is greatly increased without significant degradation of detection gain. The rest of the paper is organized as follows: We first restate the DMD in Section 2. In Section 3, we introduce the method using DMD for target detection and define some indexes for performance evaluation. Section 4 contains the results of experiments and the performance comparison among the four methods based on the low-rank and sparse theory. The conclusions are provided in Section 5.

## 2. Dynamic Mode Decomposition

DMD is an Arnoldi-like method that is based on the Koopman operator, which originally was introduced in the fluid mechanics community [23,24]. This method is usually used to calculate the eigenmodes of an approximate linear model. In this section, we restate the DMD from the perspective of the matrix decomposition, which is the basis for low-rank and sparse decomposition in the next section.

Consider the snapshot sequence, which is given by a matrix $X\in R^{M\times N}$,

(1) $$X=\left[x_{1},x_{2},\cdots ,x_{N}\right]$$

where $x_{i}\in R^{M\times 1}$ is a vector representing the *i*-th snapshot. We regard the process of *N* snapshots as an approximate linear model.

It is assumed that a Koopman operator $A\in R^{M\times M}$ connects the *i*-th snapshot to the subsequent (i+1)-th snapshot, which is:

(2) $$x_{i+1}=Ax_{i}+z_{i}$$

where $z_{i}\in R^{M\times 1}$ is the residual vector. The data can be grouped into matrices as follows:

(3) $$X_{l}=\left[x_{1},x_{2},\cdots ,x_{N-1}\right]$$

(4) $$X_{r}=\left[x_{2},x_{3},\cdots ,x_{N}\right]$$

Therefore, we can obtain Equation (5) from Equations (2)–(4).

(5) $$X_{r}=AX_{l}+Z$$

where $Z=\left[z_{1},z_{2},\cdots ,z_{N-1}\right]$ is the residual matrix.

From another perspective, suppose that the (i+1) snapshots span a linear space; therefore, the (i+1)-th snapshot can be approximated by a linear combination of the previous snapshots as follows:

(6) $$x_{N}=\alpha_{1}x_{1}+\alpha_{2}x_{2}+\cdots +\alpha_{N-1}x_{N-1}+r$$

where $r\in R^{M\times 1}$ is the residual vector. Then, Equation (6) can be written as:

(7) $$x_{N}=X_{l}\alpha +r$$

where $\alpha ={\left[\alpha_{1},\alpha_{2},\cdots ,\alpha_{N-1}\right]}^{T}$ Then, $X_{r}$ can be written as:

(8) $$X_{r}=X_{l}K+re_{N-1}^{T}$$

where $e_{N-1}$ is the (N-1)-th unit vector and $K\in R^{\left(N-1\right)\times \left(N-1\right)}$ is a companion matrix determined by:

(9) $$K=\left[\begin{array}{ccccc} 0 & 0 & \cdots & 0 & \alpha_{1}\\ 1 & 0 & \ddots & \vdots & \alpha_{2}\\ 0 & 1 & \ddots & 0 & \vdots \\ \vdots & \ddots & \ddots & 0 & \alpha_{N-2}\\ 0 & \cdots & 0 & 1 & \alpha_{N-1} \end{array}\right]$$

$X_{l}=\mathrm{QR}$ denotes the QR-decomposition of $X_{l}$, and then, the least squares solution of α in Equation (7) is solved by:

(10) $$\alpha =R^{-1}Q^{H}x_{N}$$

The residuals in Equations (5) and (8) can be ignored in the approximate linear model; thus, we obtain:

(11) $$AX_{l}=X_{l}K$$

Actually, we care more about the eigenvalues and eigenvectors of A, rather than it itself. Hence, we can reduce the computational complexity via the singular value decomposition and the eigenvalue decomposition. $X_{l}=U\Sigma V^{H}$ denotes the singular value decomposition of $X_{l}$, then:

(12) $$A=U\tilde{K}U^{-1}$$

where:

(13) $$\tilde{K}=\Sigma V^{H}K{\left(\Sigma V^{H}\right)}^{-1}$$

We can solve the eigenvalue problem by:

(14) $$\tilde{K}w_{i}=\lambda_{i}w_{i}$$

where $\lambda_{i}$ is the *i*-th eigenvalue of $\tilde{K}$ and $w_{i}$ is the corresponding eigenvector. Hence, Equation (12) can be written as:

(15) $$A=\mathrm{UW}\Lambda W^{-1}U^{-1}$$

where W is the matrix whose columns consist of $w_{i}$, and Λ is the diagonal matrix whose diagonal elements consist of $\lambda_{i}$.

In the field of fluid mechanics, the researchers focus on the eigenmodes of an approximate linear model. The eigenmodes can be represented by the eigenvalues and eigenvectors of A. Instead, we care more about how X is represented by the eigenmodes. Therefore, further work is carried out.

By ignoring the residuals, any column of X can be represented as:

(16) $$x_{i}=A^{i-1}x_{1}$$

Define Φ=UW; thus, $\Phi^{-1}=U^{-1}W^{-1}$. Define $b=\Phi^{-1}x_{1}$, then any column of X can be represented as:

(17) $$x_{i}=\Phi \Lambda^{i-1}b$$

Let i=1; thus, $b={\left[b_{1},b_{2},\cdots ,b_{k}\right]}^{T}$ is solved by $b={\left(\Phi^{H}\Phi \right)}^{-1}\Phi x_{1}$, where *k* is the number of nonzero eigenvalues of A. Notice that $\Lambda^{i-1}$ is a diagonal matrix; thus, Equation (17) can be written as:

(18) $$x_{i}=\Phi \left[\begin{array}{cccc} b_{1} & 0 & \cdots & 0\\ 0 & b_{2} & & \vdots \\ \vdots & & \ddots & 0\\ 0 & \cdots & 0 & b_{k} \end{array}\right]\left[\begin{array}{c} \lambda_{1}^{i-1}\\ \lambda_{2}^{i-1}\\ \vdots \\ \lambda_{k}^{i-1} \end{array}\right]$$

Therefore,

(19) $$X=\Phi BV_{\mathrm{and}}$$

where B is a diagonal matrix whose diagonal elements are $b_{i}$ and $V_{\mathrm{and}}$ is the Vandermonde matrix.

(20) $$V_{\mathrm{and}}=\left[\begin{array}{cccc} 1 & \lambda_{1} & \cdots & \lambda_{1}^{N-1}\\ 1 & \lambda_{2} & & \lambda_{2}^{N-1}\\ \vdots & & \ddots & \vdots \\ 1 & \lambda_{k} & \cdots & \lambda_{k}^{N-1} \end{array}\right]$$

Now, X is represented as a multiplication of three matrices using the eigenvalues and eigenvectors of A. It will be used for low-rank and sparse decomposition in the next section.

## 3. Low-Rank and Sparse Decomposition for Detection

Low-rank and sparse decomposition can be achieved by different methods. Weichang Li et al. and Feng-Xiang Ge et al. achieved this decomposition by using the random projection algorithm and convex optimization, respectively [21,22]. In this section, we use DMD to achieve low-rank and sparse decomposition and apply it for moving target detection. We also define some indexes for performance evaluation.

Consider a situation in which *N* pulses of the signal are transmitted and the beamformings of the received data are carried out. Define a sequence of matrices $D_{i}\in R^{N_{r}\times N_{\theta }}\left(i=1,2,\ldots ,N\right)$ to represent the beamforming of received data after the *i*-th pulse. $N_{r}$ and $N_{\theta }$ are the dimensions in the range and bearing directions, respectively. Define $x_{i}\in R^{M\times 1}$ as the vectorizing of $D_{i}$, where $M=N_{r}N_{\theta }$. Thus, X can be formed as Equation (1).

In general, there is a high correlation among each frame of reverberation; thus, the matrix can be decomposed as:

(21) X=L+S

where $L\in R^{M\times N}$ is a low-rank matrix that represents the coherent part of X and $S\in R^{M\times N}$ is a sparse matrix that represents the incoherent part of X. This means that L contains the coherent part of reverberation, which is the principal component of reverberation, and S contains the incoherent part of reverberation. Clearly, the echo of the moving target is uncorrelated; thus, S also contains the target. Our goal is to separate S from X to achieve the moving target detection.

Assume that $\lambda_{p}$, where p=1,2,⋯,N, satisfies $∥\lambda_{p}∥\approx 1$ and that $\lambda_{j}$, ∀j≠p is bounded away from zero; then,

(22) $$\Phi B\left(e_{p}^{T}V_{and}\right)\approx \left[\Phi_{p}b_{p},\Phi_{p}b_{p},\cdots ,\Phi_{p}b_{p}\right]$$

where $e_{p}$ is the *p*-th unit vector, $\Phi_{p}$ is the *p*-th column vector of Φ and $b_{p}$ is the *p*-th element of vector b. Obviously, it is an approximate low-rank matrix, so the two parts of X can be represented as:

(23) $$L=\Phi B\left(e_{p}^{T}V_{and}\right)$$

(24) S=X-L

In practice, L and S may be complex matrices. We only care about the energy; therefore, Equations (23) and (24) can be calculated with real-valued elements. They are:

(25) $$L=\left|\Phi B\left(e_{p}^{T}V_{and}\right)\right|$$

(26) $$S=X-\left|\Phi B\left(e_{p}^{T}V_{and}\right)\right|$$

where $\left|\cdot \right|$ is an operator that calculates the modulus of each element in the matrix. Notice that the incoherent parts of reverberation and noise are also contained in S. However, they are generally weaker than the moving target; therefore, we can reduce or remove the interference by setting a threshold for S. Sometimes, S has some negative values in its elements that do not make sense in terms of energy. We can set them equal to zero, which has no effect on the detection. Finally, we obtain the results of detection in each frame via reshaping each column of S to a matrix that is similar to $D_{i}$.

To evaluate the performance, some indexes are defined in the following. Suppose that the energy distribution of the target is $T_{i}\in R^{N_{r}\times N_{\theta }}\left(i=1,2,\ldots ,N\right)$ in the *i*-th beamforming. Define the target energy to total energy ratio before low-rank and sparse decomposition as:

(27) $${\mathrm{TTR}}_{\mathrm{before}}=\frac{{∥T_{i}∥}_{F}}{{∥D_{i}∥}_{F}}$$

where ${∥\cdot ∥}_{F}$ is the Frobenius norm; for instance, ${∥M∥}_{F}=\sqrt{\mathrm{tr}\left(M^{T}M\right)}$, $\mathrm{tr}\left(\cdot \right)$ is the trace of the matrix. Define the target energy to total energy ratio after low-rank and sparse decomposition as:

(28) $$\mathrm{TT}R_{\mathrm{after}}=\frac{{∥{T_{i}}^{\prime }∥}_{F}}{{∥{D_{i}}^{\prime }∥}_{F}}$$

where ${T_{i}}^{\prime }\in R^{N_{r}\times N_{\theta }}\left(i=1,2,\ldots ,N\right)$ is the energy distribution of the target in the *i*-th beamforming after low-rank and sparse decomposition, and ${D_{i}}^{\prime }\in R^{N_{r}\times N_{\theta }}\left(i=1,2,\ldots ,N\right)$ is the total energy distribution in the *i*-th beamforming after low-rank and sparse decomposition. Define the detection gain as:

(29) $$G=\frac{\mathrm{TT}R_{\mathrm{after}}}{\mathrm{TT}R_{\mathrm{before}}}$$

*G* is an index to evaluate the detection performance. The larger *G* indicates better detection performance. When G<1, the detection fails.

## 4. Results

We tested our method for moving target detection with three sets of underwater acoustic data and compared it with previous methods in terms of the detection gain and the computation time.

**Figure 1:**
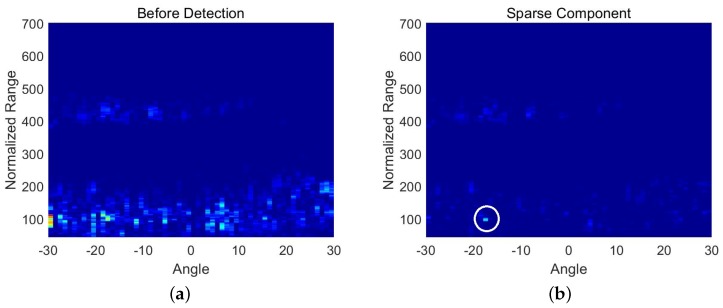

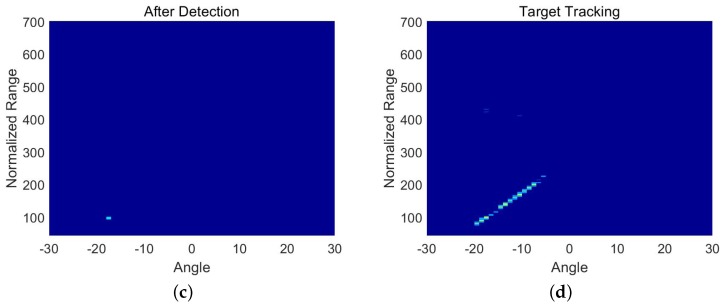
Detection results of the virtual target. (**a**) is one frame of data in beamspace before detection; (**b**) is the sparse component of the corresponding frame data; (**c**) is the detection result of corresponding frame data; (**d**) is the trajectory of the target.

The first set of data was collected in the waters near Vladivostok in Russia. The data do not contain the target echo; therefore, we added the target echo artificially. The dataset consisted of 30 frames of beamforming data. Each frame was a matrix with 658 samples along the range axis and 61 samples along the radial angle axis. The detection results are shown in Figure 1. Figure 1a is one frame of data in the beamspace before detection. It is observed that the strong reverberant background overwhelms the target signal. Figure 1b is the sparse component, which was decomposed from the corresponding frame by DMD. Most of the interference in Figure 1a has been removed, and the target (circled in white in Figure 1b) is clearly distinguishable. Figure 1c is the detection result of the corresponding frame data. It shows that the residual interference can be further removed by setting a threshold. Figure 1d is the overlap of the detection results, which shows the trajectory of the target.

**Figure 2:**
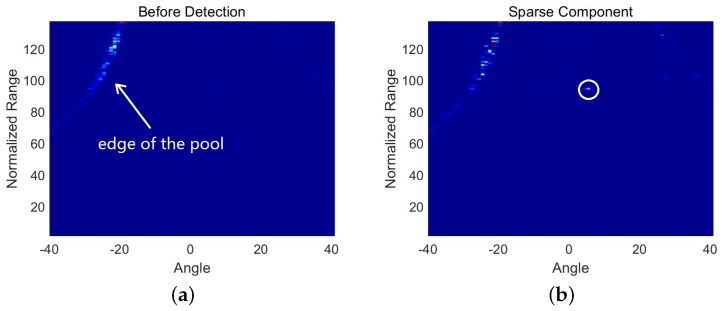

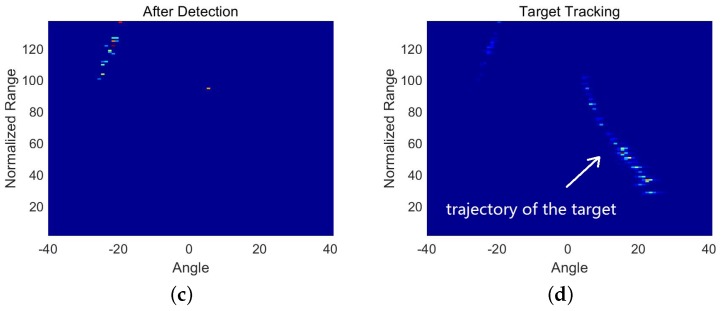
Detection results of a hollow ball. (**a**) is one frame of data in beamspace before detection; (**b**) is the sparse component of the corresponding frame data; (**c**) is the detection result of corresponding frame data; (**d**) is the trajectory of the target.

The second set of data was collected from the experimental pool of the Acoustic Science and Technology Laboratory. We dragged a hollow ball that was 20 cm in diameter to simulate a moving target. The edge of the pool was regarded as a strong interference source. The dataset consisted of 25 frames of beamforming data. Each frame was a matrix with 137 samples along the range axis and 81 samples along the radial angle axis. The detection results are shown in Figure 2. Figure 2a is one frame of data in beamspace before detection. The target cannot be observed because the interference is too strong. Figure 2b is the sparse component, which was decomposed from the corresponding frame by DMD. Most of the interference in Figure 2a has been removed so that the target (circled in white in Figure 2b) is observable. Figure 2c is the detection result of the corresponding frame data. The detection result shown in Figure 2c is similar to that shown in Figure 1c. The difference between Figure 1c and Figure 2c is that the interference in Figure 2c cannot be removed completely, because the energy of the incoherent part of the interference is not much weaker than the counterpart of the target. Across the 25 frames, the target follows a continuous trajectory, so the position of the target can still be decided.

**Figure 3:**
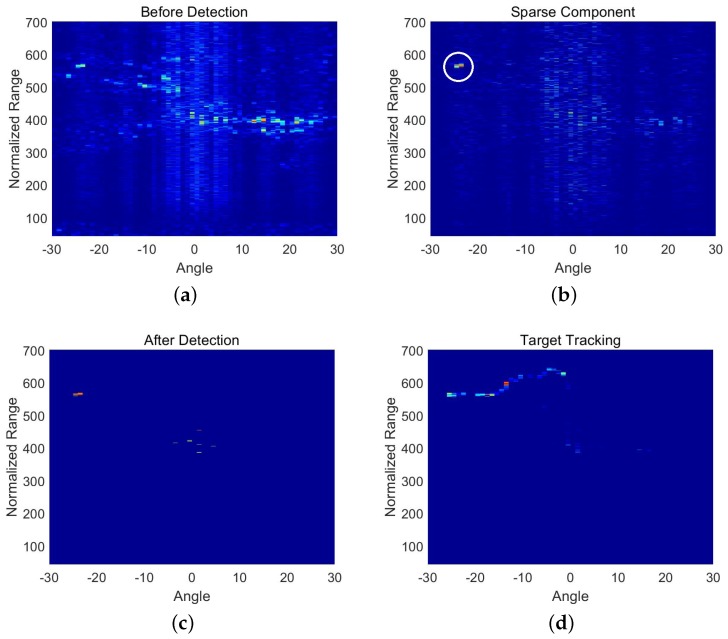
Detection results of an ROV. (**a**) is one frame of data in beamspace before detection; (**b**) is the sparse component of the corresponding frame data; (**c**) is the detection result of corresponding frame data; (**d**) is the trajectorymoving path of the target.

The third set of data was collected in the Songhua River in Harbin in China, which contained the target echo. The target was a small remotely-operated vehicle (ROV). We controlled the ROV moving horizontally such that the trajectory of the target was simple. The dataset consisted of 25 frames of beamforming data. Each frame was a matrix with 658 samples along the range axis and 61 samples along the radial angle axis. The detection result shown in Figure 3 is similar to that shown in Figure 2. The interference in Figure 3c cannot be removed completely at present, and it also needs to use the trajectory of the target to distinguish between the target and strong interference.

The content discussed in this paper is based on reverberation being the primary interference. Hence, the effect of noise on detection was not included in the performance evaluation. We evaluated the detection performance of our method on the detection gain and the computation time. Our method was compared with the sequential random projection based subspace tracking and sparse filtering (SRPSS) method and fast data projection based subspace tracking and sparse filtering (FDPSS) method proposed in [21] and the accelerated proximal gradient (APG) method proposed in [22]. The three sets of underwater acoustic data were used in the comparison.

**Figure 4:**
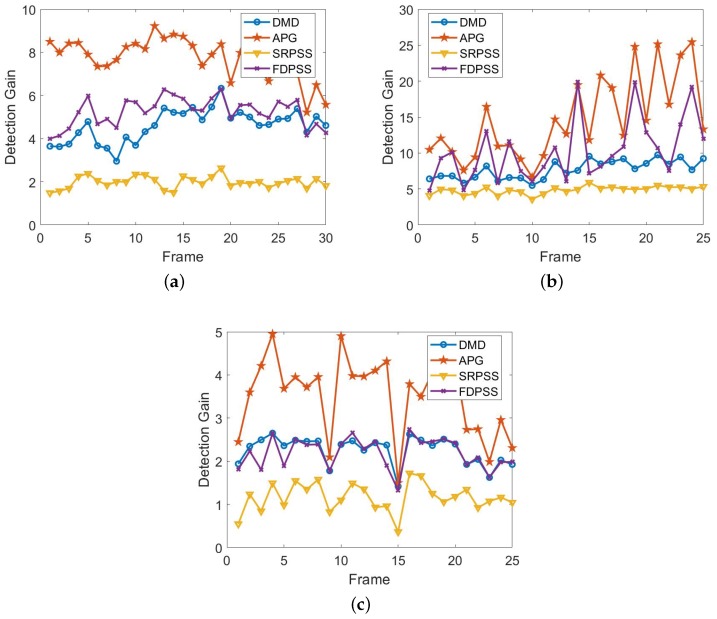
Results of detection gain. (**a**) is the result of the first set of data; (**b**) is the result of the second set of data; (**c**) is the result of the third set of data.

We calculated the detection gain of the three sets of underwater acoustic data by Equation (29). The results are shown in Figure 4. Figure 4a is the result of the first set of data. Figure 4b is the result of the second set of data. Figure 4c is the result of the third set of data. The results show that the detection gain of APG is the maximum and the detection gain of SRPSS is the minimum in the three sets of data and also show that the detection gain of FDPSS is slightly larger than that of DMD, although they may be approximately equal.

**Figure 5:**
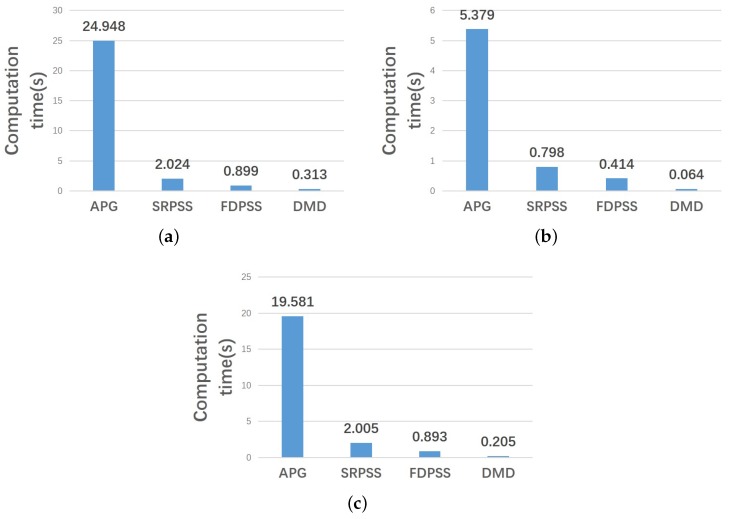
Results of calculation. (**a**) is the result of the first set of data; (**b**) is the result of the second set of data; (**c**) is the result of the third set of data.

The calculations are conducted and timed on the same workstation with an Intel(R) Core(TM) i5-4200M 2.50-GHz CPU and 8 GB RAM, running Windows 7 (64 bit) and MATLAB 2018a. The results are shown in Figure 5. Figure 5a shows the result of the first set of data. Figure 5b shows the result of the second set of data. Figure 5c shows the result of the third set of data. These results show that the DMD method is the fastest. Our method improves the computation speed by approximately 4–90 times.

From the comparison, we verified that our method greatly improved the computation at the expense of a slight loss of detection gain. However, the method is not perfect. There was still unremovable interference in the detection results. We must use the information of trajectory to separate the target from the interference. The cause of the interference should be studied in the future to remove it. It is also a good idea to apply the information of trajectory to DMD, because it may achieve greater efficiency and better detection performance.

## 5. Conclusions

In this paper, we developed a new method for moving target detection in a strong reverberant background. This work was inspired by the application of low-rank and sparse decomposition in target detection. By classifying and combining the eigenmodes after DMD, we realized the low-rank and sparse decomposition by using a different method for target detection. Compared with the random projection algorithm and convex optimization, our method has a concise and clear derivation, which makes it easier for implementation. More importantly, our method is computationally effective because no iteration is involved. The effectiveness and accuracy of our method have been demonstrated by successful moving target detection in three sets of underwater acoustic data.

## Abbreviations

The following abbreviations are used in this manuscript:

LFM	linear frequency modulation
WVD	Wigner-Ville distribution
FrFT	fractional Fourier transform
PCI	principal component inverse
DMR	dominant mode rejection
DMD	dynamic mode decomposition
ROV	remotely-operated vehicle
SRPSS	sequential random projection-based subspace tracking and sparse filtering
FDPSS	fast data projection-based subspace tracking and sparse filtering
APG	accelerated proximal gradient
